# *IARS2*-related disease manifesting as sideroblastic anemia and hypoparathyroidism: A case report

**DOI:** 10.3389/fped.2022.1080664

**Published:** 2023-01-10

**Authors:** Yan Gong, Xiao Ping Lan, Sheng Guo

**Affiliations:** ^1^ Department of Endocrinology, Shanghai Children's Hospital, School of Medicine, Shanghai Jiao Tong University, Shanghai, China; ^2^ Department of Molecular Laboratory, Shanghai Children's Hospital, School of Medicine, Shanghai Jiao Tong University, Shanghai, China

**Keywords:** CAGSSS, IARS2, sideroblastic anemia, hypoparathyroidism, mitochondrial disease

## Abstract

**Background:**

*IARS2* (EC6.1.5) is a mitochondrial isoleucine-tRNA synthetase. Despite the fact that only fewer than 30 patients have been reported in the literature, mitochondrial disorders caused by pathogenic variants in the IARS2 gene (OMIM: 616007) have a very broad and variable clinical phenotype spectrum. We present a child who has sideroblastic anemia and hypoparathyroidism as a result of a previously unreported mutation in the IARS2 gene.

**Case presentation:**

A 14-year-old girl who had been anemic for 12 years was diagnosed with pure red cell aplasia (hemoglobin 42 g/L, reference range 110–160) at the age of 2. Her anemia was resistant to high-dose intravenous gamma globulin and cyclosporine therapy and required monthly blood transfusions to maintain normal hemoglobin levels. She developed cataracts at the age of 6 and was cured by phacoemulsification. At the age of 8, she visited the endocrine department, because of mental and physical retardation accompanied by repeated convulsions, and the antiepileptic treatment was ineffective. She was diagnosed with hypoparathyroidism. To control the convulsions, she was given calcitriol orally as well as large doses of calcium supplements. Due to severe growth and development delays, delayed sexual development, and hypokinesia at the age of 13.5Y, the parents agreed to a whole-exon gene sequencing test. IARS2 gene compound heterozygous variants c.2450G > A (*p*.Arg817His) and c.2511del (*p*.Leu838Phefs*69) were discovered. The girl was then diagnosed with *IARS2*-related disease and given a cocktail therapy of coenzyme Q_10_, vitamin B_2_, L-Carnitine and vitamin E. Although the child's clinical symptoms improved, she still experienced intermittent claudication and hip joint pain. The vitamin B_6_ was discontinued after three months due to its ineffectiveness in treating anemia. Because the child's ferritin levels remained elevated, she was also prescribed long-term oral deferiprone therapy.

**Conclusion:**

Our findings broaden the clinical and genetic spectrum of *IARS2*-associated disease, and case summaries help raise clinical awareness of *IARS2*-associated disease and reduce under- and misdiagnosis.

## Introduction

Amino acid-tRNA synthetases (ARS) catalyze the attachment of amino acids to their cognate tRNAs and are required for accurate protein translation. In the cytoplasm and mitochondria, two translation pathways exist, each requiring its own set of ARS enzymes. There are 37 nuclear-encoded aminoacyl-tRNA synthetases in human cells' cytoplasm and mitochondria, 18 of which encode cytoplasmic enzymes, 17 of which encode mitochondrial enzymes, and two bilocalized synthetases (1). The nomenclature of ARS follows a systematic scheme: a single-letter code for the amino acid recognized, followed by “ARS” for cytoplasmic and bifunctional enzyme, and “2” for mitochondrial function (2). Mutations in the genes that encode the ARS enzymes frequently result in severe, early-onset recessive disease that affects a wide range of tissues and has variable clinical manifestations.

IARS2 (EC6.1.5) is a nuclear-encoded mitochondrial isoleucyl-tRNA synthetase that catalyzes the attachment of isoleucyl residues to homologous mt-tRNA in mitochondria (3). Isoleucyl-tRNA synthetase 2-associated disease was first described in 1993 in two French-Canadian cousins with clinical features similar to the previously described CAGSSS phenotype, which includes cataracts, growth hormone deficiency, sensory neuropathy, sensorineural hearing loss, and skeletal dysplasia (4). However, in 2014, a variant of *IARS2* that encodes a mitochondrial isovaleryl-tRNA synthetase was discovered (3). The clinical presentation of *IARS2*-related diseases (OMIM: 612801) varies significantly with increasing cases, revealing additional symptoms not listed in the acronym: West syndrome, Leigh syndrome, isolated cataract, and other manifestations. The phenotypic spectrum is extremely diverse.

To date, only few than 30 pathogenic/probably pathogenic *IARS2* variants have been identified in patients with CAGSSS-like phenotypes, including isolated cataracts and neurological abnormalities (4). The current study reports the detection of two heterozygous IARS2 gene variants in a Chinese patient with a clinical diagnosis of *IARS2*-associated disease using exon sequencing from the subject's whole blood genomic DNA: *p*.Arg817His missense variant inherited from the subject's mother and *p*.Leu838Phefs*69 shift variant inherited from the subject's father. Our findings broaden the range of pathogenic *IARS2* variants and the disease expression spectrum.

### Case presentation

The 14-year-old female patient has been treated for anemia for 12 years and intermittent convulsions for 11 years. The child was delivered by cesarean section in 39 weeks gestation due to intrauterine distress, birth weight: 2850 g (-1SD). She was G3P1, G1 and G2 were aborted, both parents were healthy. At 6 months, the child could sit, stand at 12 months. At 15 months the child could walk with assistance and speak simple words at 24 months. After the age of two years, the child gradually develops language regression, is unable to speak, and can only understand simple instructions. Family history was negative on hereditary and congenital diseases.

At the age of 2, the child was admitted to the hospital with a respiratory tract infection and convulsions. She was discovered to be anemic (hemoglobin 42 g/L, reference range 110–160), with poor psychomotor development, no obvious signs of skin or mucosal bleeding, no vomiting, and transient convulsive episodes following crying and fussing. Aspiration of bone marrow revealed active bone marrow proliferation, with a significantly elevated granulocyte-to-red blood cell ratio, active granulocyte proliferation, significantly decreased red lineage proliferation, and significantly increased giant lineage proliferation. Biopsy of the bone marrow reveals active granulopoiesis with suppressed red lineage proliferation. MRI of the head: brain dysplasia, abnormal basal ganglia signal on both sides.

The child was initially diagnosed with pure red blood cell aplastic anemia and was discharged after receiving high-dose intravenous gamma globulin therapy and support with red blood cell transfusion, with temporary improvement of anemia symptoms (hemoglobin 112 g/L), but the child's hemoglobin was not maintained. The hematologist suggested that the child receive cyclosporine therapy, but it did not work. The cause of the anemia could not be determined despite several bone marrow aspirations. The child still required monthly red blood cell transfusions to maintain near normal hemoglobin levels.

Due to severe vision loss, the child was diagnosed with bilateral cataracts at the age of 6, treated with cataract ultrasound emulsion surgery, and cyclosporine therapy was discontinued. When the child was 6 years old, her convulsive episodes became more frequent, increasing from 3 to 4 per year to 1–2 per month, and her hematologist advised her to see an endocrinologist. Hypocalcemia (1.26 mmol/L, reference range 2.20–2.75), hyperphosphatemia (2.41 mmol/L, reference range 0.84–1.85), and hypoparathyroidism (0.53 pmol/L, reference range 1.58–6.83) were detected at the time of consultation. Cranial CT and 3D imaging revealed bilateral lateral ventricular fullness, reduced cerebral white matter, widened sulci, and cerebral hypoplasia. The basal ganglia region had bilateral symmetrical lamellar calcium and the skull bone plate had thickened. She was diagnosed with hypoparathyroidism and was given daily oral calcium (1.5 g/d) and calcitriol (0.5 ug/d) to keep her calcium and phosphorus levels stable.

**Figure 1:**
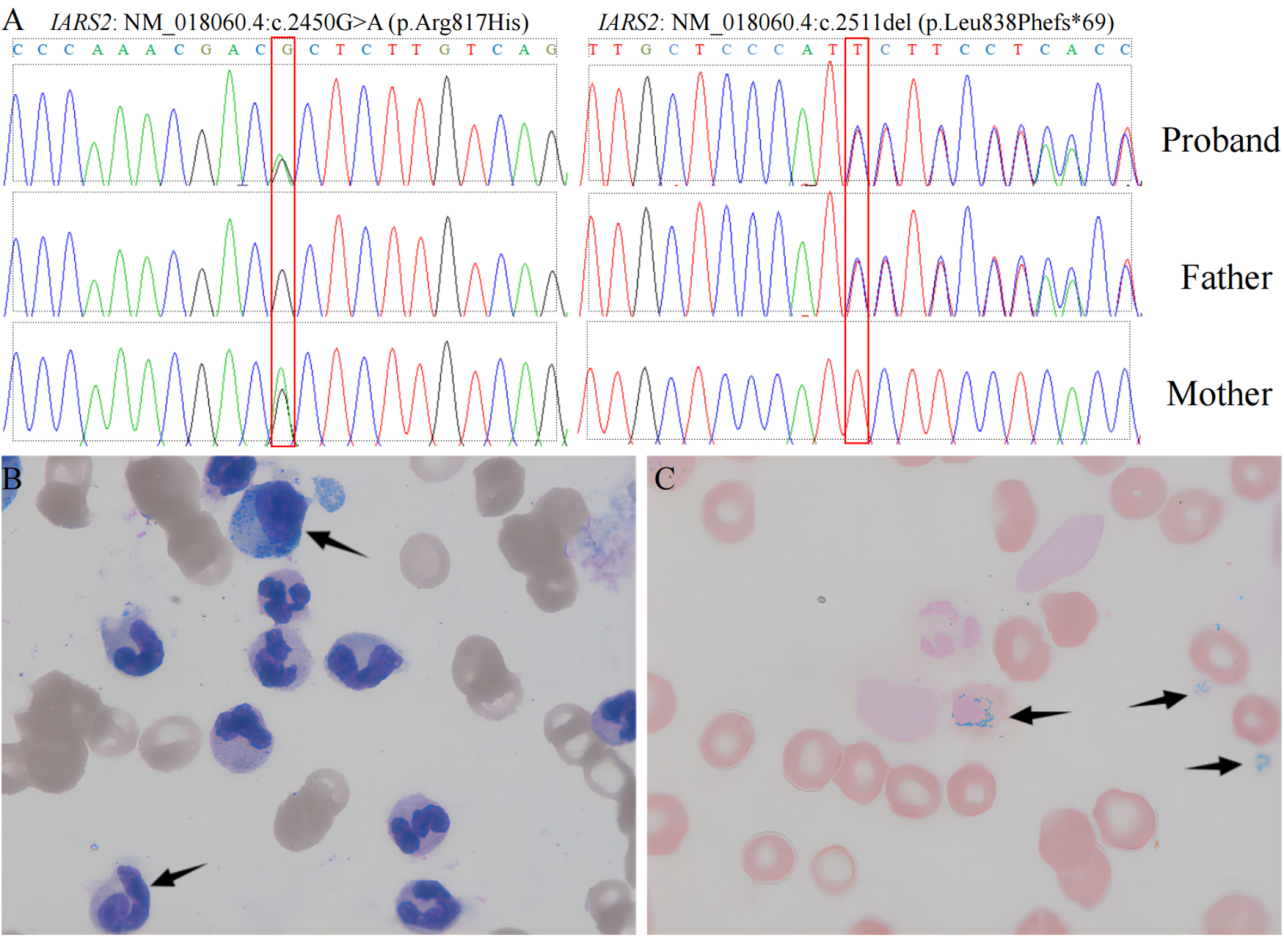
**Genomic sequencing and bone marrow testing**. Sequence electropherogram of part of exon of the IARS2 gene. left panel: IARS2: NM_018060.4:c.2450G > A (*p*.Arg817His), right panel: IARS2: NM_018060.4:c.2511del (*p*.Leu838Phefs*69). (**A**) Wright-Giemsa stained bone marrow aspirate smears: arrows (upper) denotes myelomonocytic cell, whereas arrow (lower) denote megakaryocytic cell. (**B**) Prussian-blue stained bone marrow: arrows indicate 3 of the numerous ringed sideroblasts seen.

**Figure 2:**
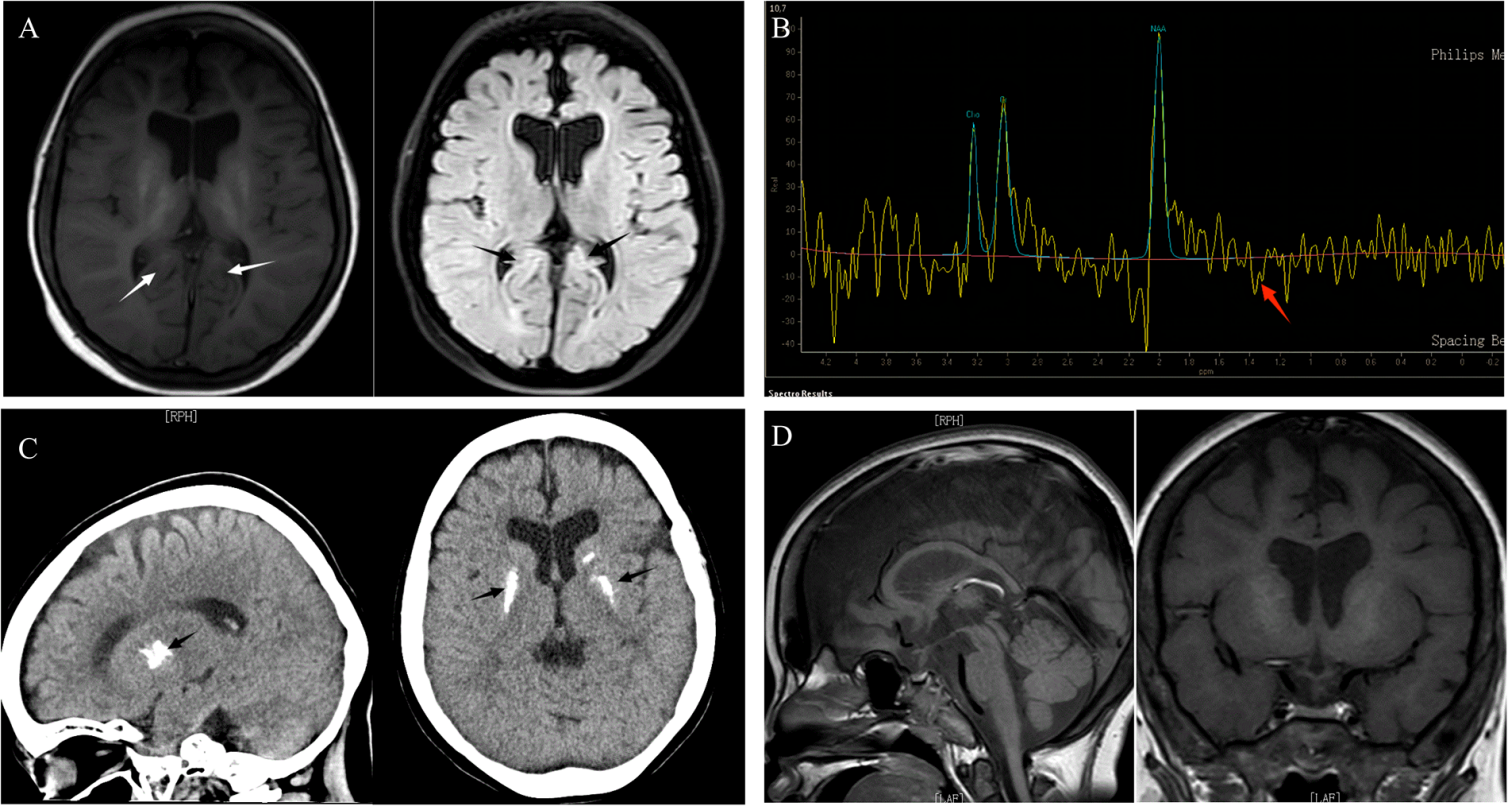
**The patient's MRI and brain CT scan findings of brain and pituitary abnormalities.** (**A**) Brain MRI: axial T1, axial T2 FLAIR: On T2-FLAIR, a strip of marginally elevated signal may be observed near to the posterior horn of both ventricles (arrows), indicating enlargement of the two ventricles and shrinkage of the brain's white matter. (**B**) MRS: mildly increased lactate peak. (**C**) CT scan: basal ganglia calcifications. (**D**) Pituitary MRI: the pituitary gland was slightly tiny, measuring 2.60 mm in height, without constriction, with uniform pituitary signal, evident strong signal in the posterior pituitary lobe, and visible pituitary stalk.

The child was free of convulsions after receiving regular calcium and calcitriol therapy, but he still had intermittent claudication and had suffered several unexpected fractures. Because the anemia did not improve, the child required monthly red blood cell transfusions, and her ferritin concentration increased and peaked at 3900 ng/ml (reference range 11–306.8 ng/ml). Concerned about the child's high ferritin level, as well as growth and metal retardation and a lack of sexual development, the patient's parents sought the advice of an endocrinologist and underwent a whole-exome gene sequencing test when she was 13.5 years old. In the subject's whole blood genomic DNA, two heterozygous IARS2 gene variants were identified: *p*.Arg817His from the subject's mother and *p*.Leu838Phefs*69 from the subject's father (Figure 1). According to the ACMG guidelines, this variant was classified as a likely pathogenic variant. The cause of the child's anemia did not support pure red blood cell aplastic anemia, and abnormal iron metabolism was clinically suspected, so we performed a bone marrow aspiration and a bone marrow iron staining test. The bone marrow test results indicated that the bone marrow was actively proliferating, with a significant increase in the ratio of granulocyte to red blood cell. The granulocyte lineage was actively proliferating, with cells of all stages visible, and the morphology was mostly normal, with visible eosinophils. The red lineage was hypoproliferative, with mid- to late-stage juvenile erythrocytes predominating, and the central bland area of mature erythrocytes was significantly enlarged. External iron staining was (+++)-(++++), internal iron staining was (-) 50%, (+) 8%, (++) 12%, (+++) 25%, (++++) 5%, and ring-shaped iron granulocytes were observed (Figure 1). Platelets are seen scattered or in clusters as the giant lineage proliferates. Since the child's intellectual and motor development, as well as his sexual development, were severely delayed, we performed an MRI of the head and pituitary gland. According to head MRI + DWI + MRS, the space outside the frontotemporal region on both sides widened, the anterior interhemispheric fissure widened, the ventricles enlarged on both sides, and white matter in the brain decreased. Strips with slightly higher signal intensity were seen on T2-FLAIR near the ventricle posterior horns on both sides, but no obvious signs were seen in the rest of the brain. MRS: NAA/Cr was 3.879 in the right basal ganglia, and Cho/Cr was 2.14, showing mildly increased lactate peak. (Figure 2). The pituitary gland is oblate and slightly small, with a height of about 2.60 mm, no restrictive elevation, uniform pituitary signal, and a high signal of the posterior pituitary lobe; the pituitary stalk is visible, not significantly widened, and centrally located. There was no significant abnormality in the visual cross section or the cavernous sinuses bilaterally. The patient's MRI and brain CT scan findings of brain and pituitary abnormalities was all showed in Figure 2. The child was diagnosed with an *IARS2-*related diseases characterized by hypoparathyroidism and sideroblastic anemia based on the results of genetic testing and bone marrow iron staining. Autosomal recessive variants of the IARS2 gene are associated with the development of cataracts, growth hormone deficiency, sensory neuropathy, sensorineural hearing loss, and skeletal dysplasia, CAGSSS (OMIM: 612801) (3). However, this child's clinical phenotype is not identical to previous reports and has a few distinguishing characteristics. The laboratory findings in this child are summarized in Supplementary material.

After being diagnosed with *IARS2*-related mitochondrial disease, the child was given mitochondrial cocktail therapy to help improve the patient's disease presentation and organ function. Coenzyme Q_10_ (10 mg/kg.d) and vitamin B_2_ (100 mg/d) promote mitochondrial energy production while also acting as antioxidants to prevent the buildup of harmful free radicals. Vitamin E (10 mg/d) is used as an antioxidant. L-Carnitine (2 g/d) aids in the transport of fatty acids and improves muscle strength and tone. To treat sideroblastic anemia, vitamin B_6_ (90 mg/d) was given to increase ALA (Aminolevulinic acid) synthase activity, iron utilization, and hemoglobin synthesis. Iron chelating agents were thought to aid iron excretion by reducing iron overload, and the child was eventually treated with deferiprone due to a suspected allergy to Desirox-500 (severe skin rash). Until now, the child had been receiving daily oral deferiprone tab (1500 mg/d), and while ferritin levels had not returned to normal, they were significantly lower than before treatment (from 3900 ng/ml to 2487 ng/ml, reference range 11–306.8). Because of the lack of significant improvement in sideroblastic anemia after three months of treatment, vitamin B_6_ was discontinued.

**Figure 3:**
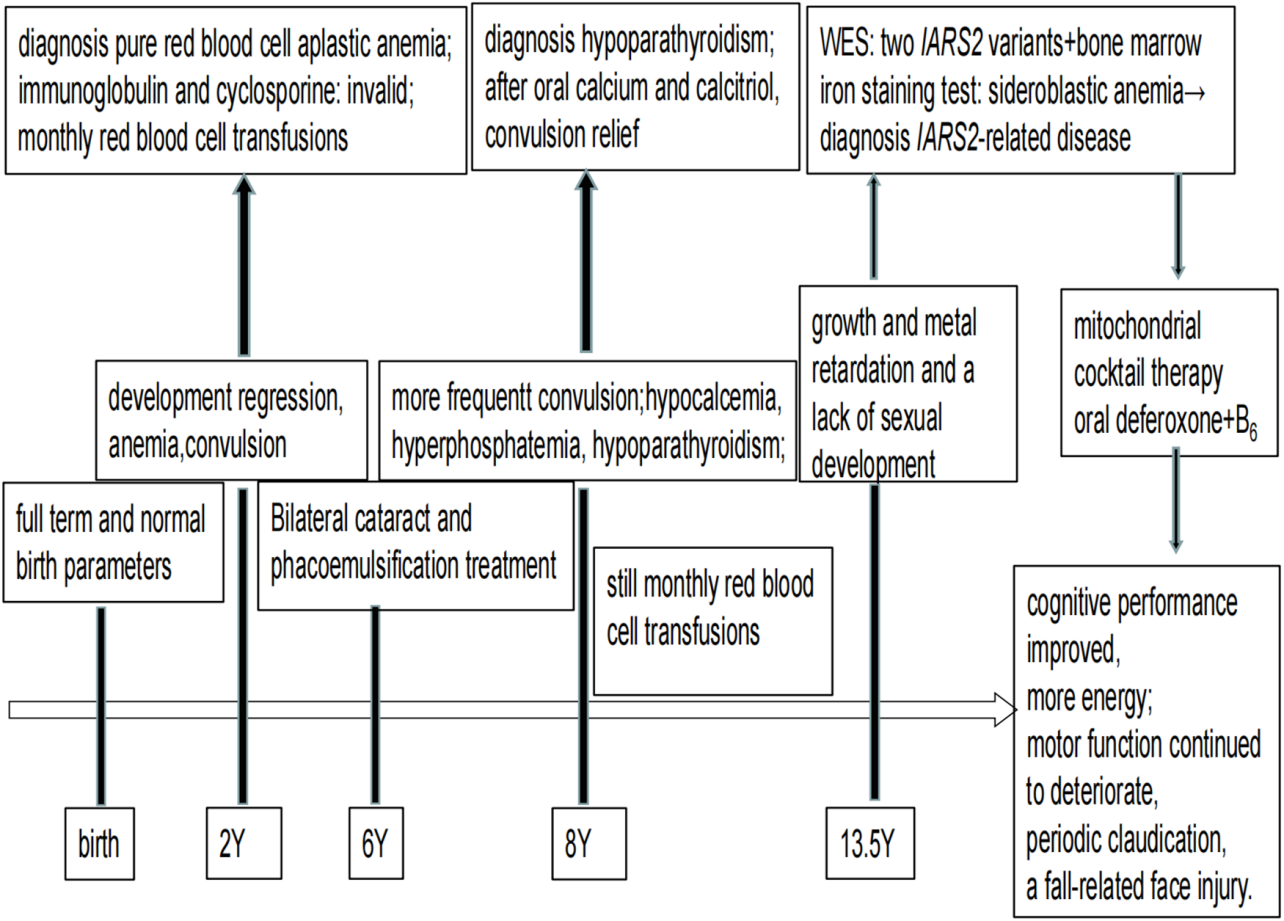
Timeline of disease progression.

At a later follow-up, we discovered that after a combination of therapies, including mitochondrial cocktail, calcitriol, calcium supplements, and behavioral and dietary instruction. Without a return of convulsions, her cognitive performance marginally improved, and she had more energy than previously. However, her motor function continued to deteriorate, she continued to experience periodic claudication, and she even experienced a fall-related face injury. Electromyography and Nerve Conduction Velocity (EMG/NCV) testing revealed no significant electromyographic changes associated with myogenic damage and motor and sensory nerve conduction velocity and amplitude normal range. The bilateral hip joints had a minor amount of fluid, but there were no indications of bone destruction, according to an MRI. It is impossible to determine whether the child is experiencing pain or vertigo because she is unable to properly convey his feelings. Timeline of disease progression are showed in the Figure 3.

### Literature review

PubMed, Embase, and Google Scholar were used to conduct our literature search. “CAGSSS”, “*IARS2*”, “*IARS2*-related disease”, “*IARS2*-related mitochondrial disease”, “aminoacyl-tRNA synthetases” were among the search phrases utilized. On December 5, 2022, 11 pertinent papers were discovered, reporting 27 pertinent cases (3–13), and including our proband, 28 cases in total. In the 28 cases of the disease, there were no racial or gender disparities. The primary clinical manifestations of the disease associated with *IARS2* were CAGSSS, isolated cataracts, or neurological abnormalities. The more prevalent phenotypes of neurological disorders are hypotonia, seizures, and developmental delay. In *IARS2*-related disease, West syndrome and Leigh syndrome are particularly prevalent, and patients frequently display a profoundly aberrant electroencephalogram (EEG) with high amplitude, chaotic spike wave patterns (hypsarrhythmia). A total of 25 variations were identified in 28 cases: 15 missense mutations, 6 stop mutations, and 4 frameshift mutations.

## Discussion

ARSs plays a role in mitochondrial tRNA biogenesis by catalyzing the attachment of amino acids to their corresponding tRNAs to form aminoacyl tRNAs (14). Pathogenic ARS gene variants cause phenotypes in tissues with high metabolic demand, particularly the central nervous system. It is still unclear how *IARS2* mutations relate to the severity and diversity of the phenotype. We can gain a deeper and more comprehensive understanding of the disease's characteristics by integrating clinical presentation, diagnostic and therapeutic processes, genotype, and prognosis. This will help us avoid misdiagnosis and underdiagnosis.

The following races have reported disease associated with *IARS2*: French-Canadian, Danish, Japanese, Scandinavian-Caucasian, Iranian, Korean, French, Sri Lankan, African-American, and Chinese. There was no clear ethnic difference in disease distribution. There were no gender differences among the 28 cases. A family history was found in nearly half of the cases. The vast majority of cases were delivered at full term, with normal birth parameters and no intrauterine growth retardation. At the time of reporting, 5 cases had died (4 due to respiratory or cardiac failure and 1 of unknown cause). 5 were adults, and 7 had average intelligence (3, 9, 12).

The mitochondrial disease itself puts the person at risk for a variety of endocrine disorders. Multiple patients with mitochondrial encephalomyopathy, lactic acidosis, and stroke-like episodes (MELAS), mtDNA deletion disorders, and nuclear encoded defects have been linked to growth hormone deficiency (GHD) (15). Short stature and GHD were initially one of the most prominent features of CAGSSS, but as the number of reported cases increased, it was reported that only almost half of individuals with *IARS2*-related diseases had short stature (13/28). Six of them were tested for growth hormone deficiency, and four were found to be deficient. Two patients received short-term growth hormone replacement therapy with good results, but no detailed treatment description is available (9). Our reported case was noticeably short in stature (-2.3 SD). Because of concerns about the child's mental retardation and delayed sexual development, the parents did not consider undergoing a growth hormone stimulation test to assess growth hormone levels. Adrenal insufficiency has been identified in a number of patients with other types of mitochondrial dysfunction (16). Hypoglycemia may be associated with adrenal insufficiency in *IARS2*-related diseases. 2 cases of hypoglycemia among the available cases were linked to adrenal insufficiency, while the other 2 cases of transient hypoglycemia had an unknown cause (9). Our patient had normal ACTH (adrenocorticotropic hormone) (6.37 pmol/L, 1.59–14 pmol/L) and cortisol (201.87 nmol/L, 73–312 pmol/L), and no hypoglycemia occurred.

Our study and three other studies (11, 12, 13) reported a total of 4 patients with hypoparathyroidism, which enhancing the spectrum of endocrine system clinical manifestations. The most accepted pathophysiological mechanism of hypothalamic-pituitary axis dysfunction is associated with endocrine glands' high energy demands; impaired mitochondrial ATP production and/or oxidative stress may greatly reduce the ability to secrete hormone or maintain normal feedback (15). Differences in energy requirements between tissues result in differences in affected organs, resulting in differences in clinical manifestation severity (17). Only 16/28 *IARS2*-related individuals exhibited high lactate levels in their blood or CSF fluid or lactate spectrum findings on an MRI, whereas 14/28 had abnormalities on their brain scans. Five of the 25 patients in another study who had *ARS2* mutations (3 *IARS2*, 1 *YARS2*, and 1 *FARS2*) had normal brain MRI images (13). As a result, normal lactate and brain MRI images do not rule out the possibility of *ARS2* mutations in mitochondrial diseases.

Sideroblastic anemia has been reported in 6 current cases of *IARS2* including our report (4). Congenital sideroblastic anemia (CSA) is a type of sideroblastic anemia caused by a mutation in an iron metabolism-related gene. It is divided into three types based on pathophysiology: mutations in genes involved in heme synthesis, iron-sulfur (Fe-S) cluster synthesis and transportation, and mitochondrial respiratory chain synthesis (18). Mitochondria are the sites of heme and iron-sulfur cluster synthesis, as well as the regulatory center of iron metabolism throughout the cell. Anything that inhibits heme or iron-sulfur cluster synthesis can cause mitochondrial iron overload, resulting in sideroblast rings. The ARS gene has been linked to iron metabolism and iron deposition in mitochondria (19): *YARS2* inhibits protein synthesis by interfering with tRNA modification; LARS2 gene variant causes mitochondrial respiratory chain abnormalities. It is currently unknown how *IARS2* interferes with iron metabolism. Current treatments, such as iron chelators Defetoxamine (DFO) and Deferasirox; antioxidants vitamin E and Coenzyme Q_10_, are primarily aimed at oxidative damage and decreased mitochondrial function caused by iron accumulation. Our patient was given Deferiprone in the hopes of reducing iron overload; while ferritin levels did not return to normal, they were significantly lower than before treatment. Her cognitive function and vitality improved after receiving the mitochondrial cocktail. However, her condition is still progressing slowly; she is still regressing in motor function, lagging in mental development, can not speak, requires diapers, has not begun pubertal development, and experiences intermittent claudication and even accidental falls.

Cardiac involvement is common in pediatric mitochondrial disease patients. Cardiomyopathy (dilated and hypertrophic cardiomyopathy), conduction abnormalities, and ventricular dysfunction are common cardiovascular manifestations of mitochondrial disease (20). However, only 5 of 28 cases with *IARS2* mutations had cardiac issues (3 hypertrophic cardiomyopathy, 1 mild ventricular hemorrhage, and 1 Wolff-Parkinson White syndrome). Patients with mitochondrial disease are at risk for cardiac abnormalities and sudden cardiac death. Patients with *IARS2*-related mitochondrial disease should have an ECG and echocardiogram performed on a regular basis. Our patient's cardiac ultrasound revealed no significant intracardiac structural abnormalities, and left ventricular systolic function was normal.

In the 28 reported cases, 25 variants were discovered: 15 missense mutations, 6 stop mutations, and 4 frameshift mutations. The mitochondrial leader peptide, tRNA synthetase domain, anticodon binding domain, and Zinc finger domain were all involved in the IARS2 gene. In the recent literature, they identified 4 homozygous variants: *p*.His761Arg (2 patients), *p*.Gly874Arg (1 patient), *p*.Pro909Ser (1 patient), and *p*.Pro909Leu (3 patients) (4). These patients all fit the CAGSSS profile, since they were intelligent and self-sufficient. And these 4 homozygous missense pathogenic variants were all found near the anticodon-binding domain. As a result, we hypothesized that homozygous mutations in the anticodon-binding domain could be linked to the CAGSSS phenotype, average intelligence, and a favorable prognosis. There are currently only 7 cases reported with the CAGSSS phenotype, and all of them are homozygous mutations (4). As a result, it is unclear whether compound heterozygous mutations in the same domain have similar phenotypes, which needs to be confirmed by more case reports.

Among patients with *IARS2*-related disease, 4 cases of hypoparathyroidism and 6 cases of sideroblastic anemia have been reported. However, we are the first to report an *IARS2*-related disease with hypoparathyroidism and sideroblastic anemia as the main characters. Furthermore, the two heterozygous IARS2 gene variants that caused this case are reported for the first time: *p*.Arg817His and *p*.Leu838Phefs*69. The variant (*p*.Arg817His) has been reported as compound heterozygous in multiple Leigh syndrome patients and co-segregated with the disease in one family. According to the gnomAD database, the allele frequency of this variant in the total population is currently less than 0.01% (4/282786), with no reported pure matches. According to ACMG guidelines, this variant is classified as a suspected pathogenic variant. A 1-base pair deletion results in an out-of-frame transcription product and an early termination codon in the shift variant (*p*.Leu838Phefs*69). This variant may result in protein truncation or nonsense-mediated mRNA degradation, resulting in the loss of function of the IARS2 gene's protein product. Although this variant has not been reported in the literature, other truncating variants of this gene have been linked to Leigh syndrome. In this case, this variant was discovered in trans alignment with a known pathogenic variant. The variant is not currently included in the gnomAD database. According to the ACMG guidelines, this variant is classified as a suspected pathogenic variant.

To summarize, the clinical phenotypic spectrum of *IARS2*-related diseases is very complex and broad, and the diagnosis is easily missed by relying solely on neuroimaging, conventional biochemical, and metabolite mass spectrometry tests. Because of the disease's clinical and genetic heterogeneity, efforts to uncover the intrinsic link between genotype and phenotype and treatment prognosis can help improve diagnosis and treatment and reduce misdiagnosis and mistreatment. Our findings will help to broaden the clinical phenotypic spectrum of *IARS2*-related diseases and improve understanding of IARS2 gene variants.

## Data Availability

The original contributions presented in the study are included in the article/**Supplementary Material**, further inquiries can be directed to the corresponding author/s.
