# Supplementary figures and images for: IARS2-related disease manifesting as sideroblastic anemia and hypoparathyroidism: A case report
Source: Front Pediatr. 2023 Jan 10;10:1080664. doi: 10.3389/fped.2022.1080664 (PMC9871752; doi:10.3389/fped.2022.1080664)

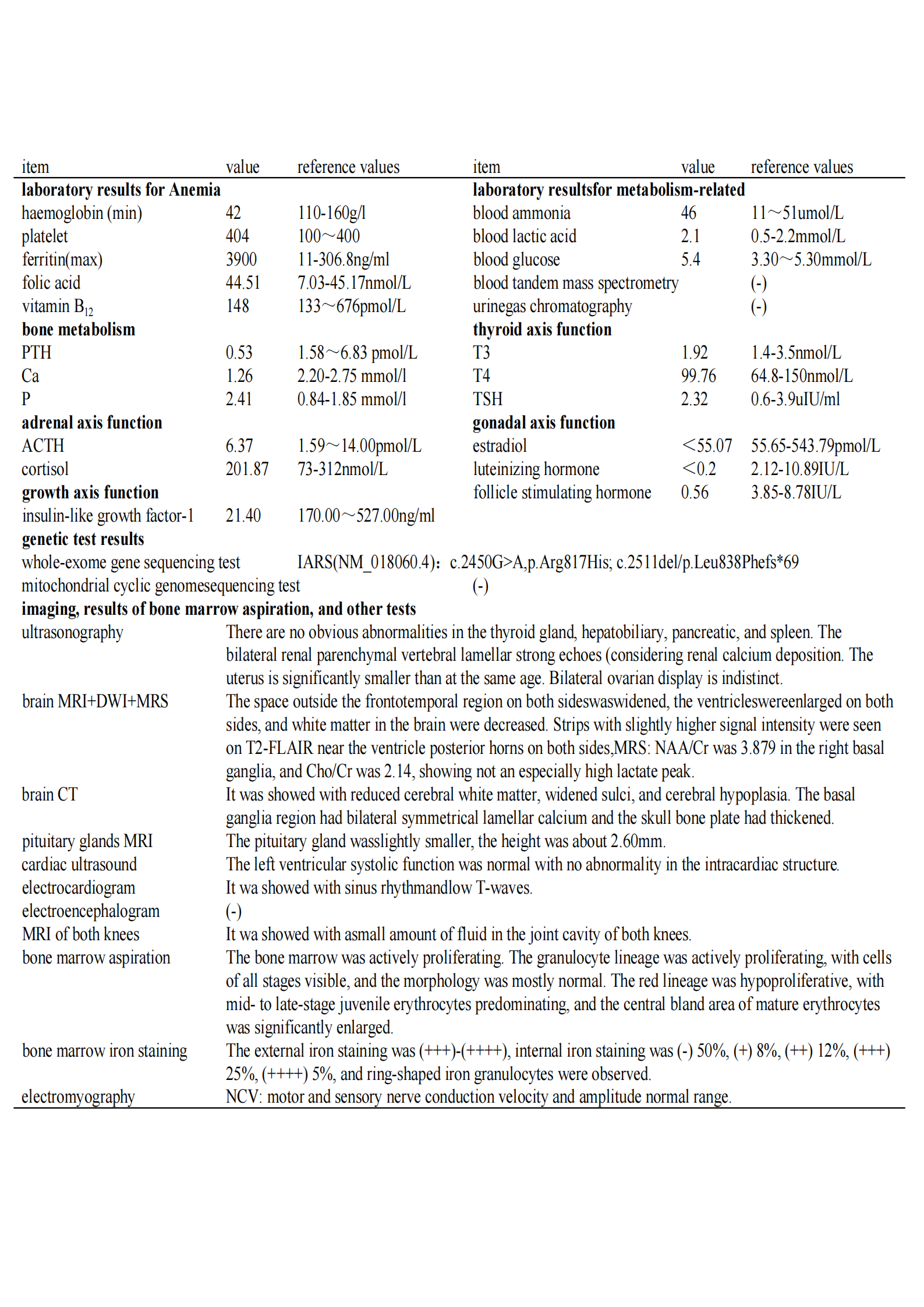

Supplement: Supplementary file 1 [file Image1.tif]
